# Comprehensive analysis of cuproptosis-related genes and tumor microenvironment infiltration characterization in breast cancer

**DOI:** 10.3389/fimmu.2022.978909

**Published:** 2022-10-20

**Authors:** Shaoran Song, Miao Zhang, Peiling Xie, Shuhong Wang, Yaochun Wang

**Affiliations:** ^1^ Center for Translational Medicine, The First Affiliated Hospital of Xi’an Jiaotong University, Xi’an, China; ^2^ The Key Laboratory for Tumor Precision Medicine of Shaanxi Province, The First Affiliated Hospital, Xi’an Jiaotong University, Xi’an, China; ^3^ Department of Breast Surgery, The First Affiliated Hospital of Xi’an Jiaotong University, Xi’an, China; ^4^ Department of Oncology, The First Affiliated Hospital of Xi’an Jiaotong University, Xi’an, China

**Keywords:** cuproptosis, breast cancer, cancer molecular subtypes, tumor microenvironment, immunotherapy

## Abstract

**Background:**

Cuproptosis is a newly discovered programmed cell death dependent on overload copper-induced mitochondrial respiration dysregulation. The positive response to immunotherapy, one of the most important treatments for invasive breast cancer, depends on the dynamic balance between tumor cells and infiltrating lymphocytes in the tumor microenvironment (TME). However, cuproptosis-related genes (CRGs) in clinical prognosis, immune cell infiltration, and immunotherapy response remain unclear in breast cancer progression.

**Methods:**

The expression and mutation patterns of 12 cuproptosis-related genes were systematically evaluated in the BRCA training group. Through unsupervised clustering analysis and developing a cuproptosis-related scoring system, we further explored the relationship between cuproptosis and breast cancer progression, prognosis, immune cell infiltration, and immunotherapy.

**Results:**

We identified two distinct CuproptosisClusters, which were correlated with the different patterns between clinicopathological features, prognosis, and immune cell infiltration. Moreover, the differences of the three cuproptosis-related gene subtypes were evaluated based on the CuproptosisCluster-related DEGs. Then, a cuproptosis-related gene signature (PGK1, SLC52A2, SEC14L2, RAD23B, SLC16A6, CCL5, and MAL2) and the scoring system were constructed to quantify the cuproptosis pattern of BRCA patients in the training cohort, and the testing cohorts validated them. Specifically, patients from the low-CRG_score group were characterized by higher immune cell infiltration, immune checkpoint expression, immune checkpoint inhibitor (ICI) scores, and greater sensitivity to immunotherapy. Finally, we screened out RAD23B as a favorable target and indicated its expression was associated with breast cancer progression, drug resistance, and poor prognosis in BRCA patients by performing real-time RT-PCR, cell viability, and IC50 assay.

**Conclusions:**

Our results confirmed the essential function of cuproptosis in regulating the progression, prognosis, immune cell infiltration, and response to breast cancer immunotherapy. Quantifying cuproptosis patterns and constructing a CRG_score could help explore the potential molecular mechanisms of cuproptosis regulating BRCA advancement and provide more effective immunotherapy and chemotherapy targets.

## Introduction

Breast cancer (BRCA) is one of the most common malignant cancers among women worldwide, with a high incidence and recurrence rate (1, 2). According to the latest statistics from 2022, breast cancer alone accounts for nearly one-third of all new cancer diagnoses (287,850 new cases) in women in the United States (3). Despite diagnostic and therapeutic strategies that have taken into consideration the heterogeneity of breast cancer (4), there are presently insufficient strategies to improve the prognosis of recurrence-free survival (RFS) and overall survival (OS) in breast cancer. At the same time, the resistance of breast cancer patients to chemotherapy, radiotherapy, or endocrine therapy makes maintaining their long-term survival an urgent challenge. In recent years, attention has been focused on the role of the tumor microenvironment (TME) in regulating breast cancer progression and prognosis and the effect of immunotherapy in breast cancer treatment (5–7). However, there is still a lack of sensitive immune-related diagnostic and therapeutic targets for breast cancer.

Cuproptosis, unlike apoptosis, ferroptosis, pyroptosis, and necroptosis, is a kind of non-apoptotic programmed cell death induced by the accumulation of intracellular copper (8, 9). Direct binding to lipid-acylated mitochondrial proteins of the tricarboxylic acid (TCA) cycle to aggregate them, followed by proteotoxic stress, is the crucial mechanism for the initiation of cuproptosis. Previous studies have illustrated the relationship between copper homeostasis and human diseases, including Wilson disease and other neurological copper disorders (10, 11), cancers (12–15), abnormal fetal development (16), and so on. However, there are no studies on the association between the newly defined cuproptosis and breast cancer oncogenesis, immune microenvironment, or immunotherapy. Therefore, exploring the physiological and pathological activities associated with cuproptosis, elucidating its underlying mechanisms affecting breast cancer progression, and identifying sensitive and effective targets for breast cancer diagnosis and treatment are crucial for early detection, diagnosis, and treatment of breast cancer.

In this study, a comprehensive assessment of the expression profile of 12 cuproptosis-related genes in breast cancer was performed to comprehensively analyze the role of CRGs on TME and immunotherapy. First, the BRCA patients in the training cohort were stratified into two cuproptosis-related clusters based on CRGs expression levels. The differentially expressed genes (DEGs) in these two clusters were then used to classify the patients into three cuproptosis-related gene subtypes. Further, differentially expressed genes with prognostic significance were used to construct a cuproptosis-related gene signature (PGK1, SLC52A2, SEC14L2, RAD23B, SLC16A6, CCL5, and MAL2) and scoring system. Three independent external testing cohorts also confirmed the stability and reliability of the scoring system. We used the cuproptosis-related gene score (CRG_score) to classify patients into high and low-CRG_score subgroups to predict overall survival (OS) and the immune landscape in BRCA, thus accurately predicting the patient long-term prognosis and response to immunotherapy. Finally, RAD23B was selected as a valuable target for *in vitro* experimental validation.

## Materials and methods

### Data acquisition and preprocessing

The gene expression profile cohort and its corresponding clinical data of BRCA patients were obtained from The Cancer Genome Atlas (TCGA) (https://portal.gdc.cancer.gov/) and the Gene Expression Omnibus (GEO) (https://www.ncbi.nlm.nih.gov/geo/) databases. Specifically, the training cohort consisted of the BRCA dataset of TCGA (113 standard samples and 1091 BRCA samples) and GSE20685 (327 BRCA samples) (**Table S1**). The testing cohorts consisted of the GSE7390 (198 BRCA samples), the GSE58812 (107 BRCA samples), and the GSE42568 (104 BRCA samples). All the gene expression data were fragments per kilobase million (FPKM) and transformed into transcripts per kilobase million (TPM) values for further analysis using the R language (version 4.1.2), the “edge” R package (Storey et al., 2021, R package version 2.26.0). Reducing the batch impact induced by non-biotechnological variations, was achieved by using the “ComBat” method in the “SVA” R package (Leek et al., 2021, R package version 3.42.0.).

### Construction of CuproptosisClusters and PCA analysis

The 12 cuproptosis-related genes, including FDX1, LIPT1, LIAS, DLD, DBT, GCSH, DLAT, PDHA1, PDHB, SLC31A1, ATP7A, ATP7B, were retrieved from previously published literature (8). To identify different cuproptosis patterns in BRCA, we performed consensus classification using the “ConsensusClusterPlus” R package (17). The tendency and smoothness of the cumulative distribution function (CDF) curve were used to figure out the clustering number (17). Principal component analysis (PCA) was conducted with the help of the function “prcomp” in the R package “stats” (R Core Team, 2021).

### Clinical characteristics and prognosis analysis in different CuproptosisClusters

Utilizing the “survival” (Therneau et al., 2021, R package version 3.2-13) and “survminer” R packages (Kassambara et al., 2021, R package version 0.4.9.), we conducted the Kaplan–Meier plot to estimate the prognostic values of BRCA patients in different molecular subtypes (18, 19). Clinical features (age, stage T, and stage N) were also compared among molecular subtypes.

### Gene set variation analysis in different CuproptosisClusters

Using the “GSVA” R package (20), the gene set variation analysis (GSVA) was conducted to estimate the differences in biological processes responsible for the characteristic patterns of cuproptosis (20, 21).

### Estimation of TME in different CuproptosisClusters

By performing the “estimate” R package (Yoshihara et al., 2016, R package version 1.0.13/r21.), the immune score of every BRCA sample was evaluated in the ESTIMATE algorithm (22). Furthermore, the fractions of 23 human immune cell subsets in the TME of different BRCA molecular subtypes were also estimated using a single-sample gene set enrichment analysis (ssGSEA) algorithm (23–26). The expression of 32 critical immune checkpoints retrieved from previous research was compared in different BRCA molecular subtypes (27).

### Identification of DEGs in different CuproptosisClusters, functional analysis, and construction of gene subtypes

We identified 968 DEGs among the different CuproptosisClusters using the “limma” package (28) in R with a fold-change of 6 and an adjusted *p < 0.001*. The functional analysis (GO and KEGG) was conducted on the DEGs using the “clusterprofiler” R package (17, 29, 30). The gene set file (c2.cp.kegg.v7.2.symbols.gmt) was obtained from the MSigDB database (https://www.gsea-msigdb.org) (30, 31). To investigate the molecular function of these cuproptosis-related DEGs mentioned above, we performed survival analysis and picked out 25 DEGs with significant prognostic values (*p* < 0.001) for further study (**Table S8**) (28, 32). Then, consensus classification was performed using the ConsensusClusterPlus” R package based on the 25 prognostic genes to divide patients into three gene subtypes (**Figure S2**, k = 3; gene subtypes A, B, and C).

### Development and validation of the cuproptosis-related gene model and CRG_score

We performed the LASSO Cox regression model using the “glmnet” R package (33, 34) to filter down the candidate cuproptosis-related genes. Finally, the 5 genes and their coefficients were kept. We obtained the penalty parameter (λ) according to the minimum criteria. After standardizing the data from the training cohort by the “scale” R package (Hadley Wickham and Dana Seidel, 2022, R package version 1.2.0.), the CRG_score was calculated as follows:

CRG_score = (0.00523335734652904 * PGK1) + (0.0185186220360186 * SLC52A2) − (0.0261649532232335 * SEC14L2) + (0.0129284730002406 * RAD23B) − (0.0297061289632435 * SLC16A6) − (0.0142610013224485 * CCL5) + (0.0014557255170858 * MAL2).

We also calculated the CRG_score of the testing cohorts using the same formula. We divided the patients from the training and testing cohorts based on the median CRG_score, and the high- and low-CRG_score groups. We performed the Kaplan–Meier analysis of overall survival using “survival” and “survminer” R packages, and ROC curve analysis using the “timeROC” R package (35). Then the calibration plots of the nomogram were executed to predict the prognosis value between the predicted 3-, 5-, and 8- or 10-year survival events and the virtually observed outcomes. Lastly, a stratified analysis was done to see if the CRG_score could still predict in different subgroups of age (65 or 65), stage T (T1-2 or T3-4), and stage N (N0 or N1-3).

Finally, we reduced the batch impact of testing cohorts (GSE7390, GSE58812, and GSE42568; n = 409) using “ComBat” method. By performing the PAM50 algorithm (36) with the “genefu” R package (37), we then assessed the molecular subtypes for each patient from testing cohorts. Samples were classified into the normal-like (n = 13), basal-like (n = 126), HER2+ (n = 57), luminal A (n = 111), and luminal B (n = 102) subtypes (**Table S2**). Each subtype was then used as an independent external validation cohort and CRG_score was calculated respectively. Survival and ROC curve analysis were then performed, as mentioned above.

### Analysis of chemotherapeutic drugs effects in high-and low-CRG_score groups

The semi-inhibitory concentration (IC50) values of chemotherapeutic drugs in high- and low-CRG_score groups were calculated using the “pRRophetic” R package (Paul Geeleher, 2014, R package version 0.5.).

### Analysis of protein expression in clinical specimen

The Human Protein Atlas (HPA) database contains sections from 46 normal human tissues and over 20 human cancers labeled with antibodies targeting more than 11000 human proteins (38). Based on the laser power and detector gain parameters used for image acquisition, combined with the image’s visual appearance, the staining intensity is rated as negative, weak, moderate and strong (39). The scoring method of protein expression is the same as described previously (40).

### Cell culture and transfection

Our human breast cancer cell lines were obtained from the Shanghai Institute of Biochemistry and Cell Biology, including MCF10A, SUM-159, MDA-MB-231, BT549, and MCF7 cells. MCF10A and SUM159 cells were cultured in DMEM/F12 (1:1) medium, MDA-MB-231 and MCF7 cells were cultured in a DMEM medium, and BT549 cells were cultured in an RPMI-1640 medium, with all recommended supplements, respectively. All cells were cultured at 37°C in a humidified incubator in an atmosphere of 5% CO_2_.

SUM-159 and MCF7 cells were transfected with corresponding siRNAs using Lipofectamine 8000 (#C0533, Beyotime, Nanjing, China) following the manufacturer’s protocol. The RAD23B siRNA constructs and a negative control siRNA were as follows: RAD23B-1, 5’- CUCCAGCAUCAGCGACAGCAUTT −3’ and 5’- AUGCUGUCGCUGAUGCUGGAGTT −3’; RAD23B-2, 5’- AGAAGCUGGAAGUGGUCAUAUTT −3’ and 5’- AUAUGACCACUUCCAGCUUCUTT −3’; NC siRNA, 5’- UUCUCCGAACGUGUCACGUdTdT −3’ and 5’- ACGUGACACGUUCGGAGAAdTdT −3’.

### Cuproptosis cell model construction and mRNA expression analyses

To promote the occurrence of Cuproptosis (8), SUM-159 and MCF7 cells were treated with 100 nM elesclomol (+1 µM CuCl2 in medium) for a 2-hour-pulse. After 24 h, cells were harvested and lysed. The real-time RT-PCR was performed as previously described (40). All primers were synthesized (Sangon Biotech, Shanghai, China) and listed in **Table S3**.

### Cell viability and IC50 assay

After transfection for 48h, SUM-159 and MCF7 cells (5 × 10^3^ cells/well) were loaded on a 96-well plate and cultivated for 0 h, 24 h, 48 h, and 72 h for cell viability assays or 48 h for the IC50 assay of Paclitaxel (0, 0.125, 2.5, 5, 10, 20 µM/L). After incubation with 20 μL of 3-(4.5-Dimethylghiazol-2-yl)-2,5-diphenyltetrazolium Bromide (MTT; 5 mg/mL; Absin Bioscience, Shanghai, China; Catalog no. abs50010) for 4 h at 37°C, the culture medium was removed, and 150 μL dimethyl sulfoxide (DMSO; Sigma, St. Louis, MO, USA) was added. Afterward, the cells were shaken for 15 min in the dark, and the optical density (OD) at 490 nm was measured using a Benchmark microplate reader (Bio-Rad, Hercules, CA, USA).

### Statistical analysis

All the data analysis was conducted by R (version 4.1.2) and *in vitro* experimental data were analyzed with the GraphPad Prism 5 (GraphPad Software, Inc., La Jolla, CA, USA). The Log-rank test, Spearman test, Wilcoxon test, Student’s t-test, and Two-way ANOVA tests were applied in this study. *p < 0.05* was considered as significant. P-values were adjusted to control for the false discovery rate (FDR) using the Benjamini-Hochberg method (41). Each experiment was done in triplicate and repeated at least three times.

## Results

### The landscape of cuproptosis-related genes in BRCA

**Figure 1:**
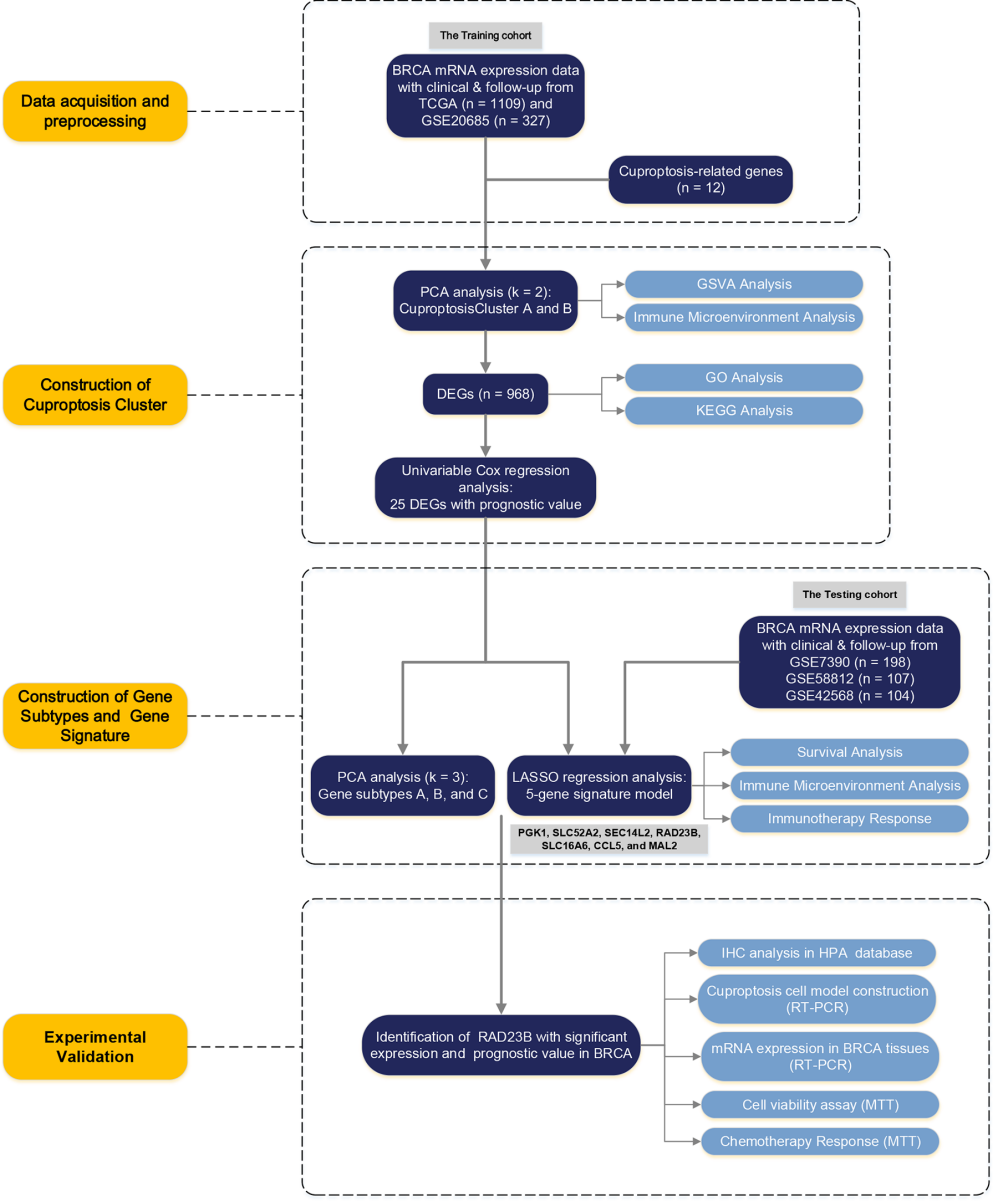
The Flowchart of the Study Design.

**Figure 2:**
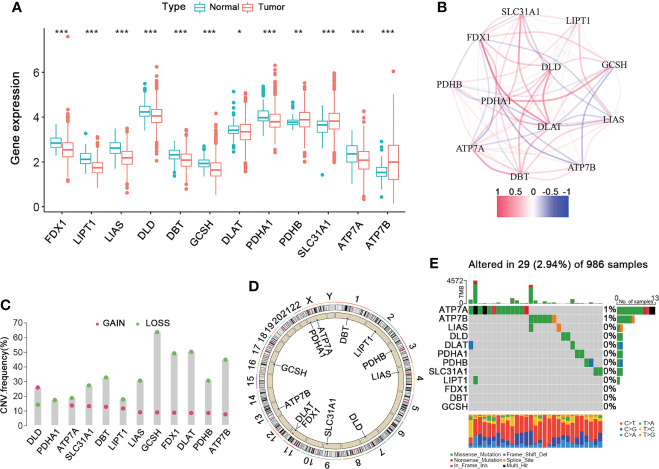
The Landscape of Cuproptosis-Related Genes in BRCA. **(A)** The gene expression levels of CRGs in BRCA compared to normal tissue (Wilcoxon test; blue: normal; red: BRCA). **(B)** The correlation network of the 12 CRGs (red: positive correlation; blue: negative correlation). **(C)** The frequency of CNV variation in CRGs (green: CNV deletion; red: CNV amplification). **(D)** The location of the CNV alteration of the CRGs changes on 23 chromosomes. **(E)** The genetic alteration on a query of CRGs. CRGs, cuproptosis-related genes; BRCA, breast cancer; CNV, copy number variant. *p < 0.05, **p < 0.01, ***p < 0.001.

**Figure 1** shows the overall design and flow chart of this study. The expression of twelve cuproptosis-related genes (CRGs) was obtained from previous studies (8). By performing the “limma” package, we analyzed the mRNA expression of CRGs based on the data of 113 normal and 1091 BRCA tissues from TCGA. As shown in **Figure 2A**, the transcriptional levels of DLAT, PDHB, SLC31A1, and ATP7B were significantly higher in the BRCA tissues than in the normal tissues. At the same time, FDX1, LIPT1, LIAS, DLD, DBT, GCSH, PDHA1, and ATP7A were significantly lower. Furthermore, we developed the correlation network containing twelve CRGs in **Figure 2B** (red: positive correlations; blue: negative correlations). Then, univariable Cox regression analysis also showed significant differences in overall survival between patients with high or low expression of the CRGs (**Table S4**). Specifically, LIPT1 (HR = 0.79, 95% CI: 0.65–0.95, *p < 0.01*), PDHB (HR = 0.97, 95% CI: 0.94–1.00, *p < 0.01*), and ATP7B (HR = 0.96, 95% CI: 0.93–1.00, *p < 0.01*) were “protective” factors for BRCA patients with HR < 1, while the DLAT (HR = 1.06, 95% CI: 1.02–1.09, *p < 0.01*), SLC31A1 (HR = 1.03, 95% CI: 1.01–1.05, *p < 0.01*), DBT (HR = 1.05, 95% CI: 0.94–1.18, *p < 0.01*), PDHA1 (HR = 1.01, 95% CI: 0.99–1.03, *p < 0.05*), ATP7A (HR = 1.00, 95% CI: 0.93–1.09, *p < 0.05*), and DLD (HR = 1.02, 95% CI: 0.99–1.04, *p < 0.05*) were “risk” factors with HR > 1.

To explore their mutation landscape, the single nucleotide variation (SNV) and copy number variation (CNV) data were downloaded from the TCGA database. Moreover, CNVs were prevalent and mostly involved deletion, though DLD had a high frequency of amplification (**Figure 2C**). **Figure 2D** shows the locations of the CNV alterations in the CRGs on their respective chromosomes. Next, we analyzed the incidence of somatic mutations in these 12 CRGs, which showed that 29 (2.94%) of the 986 BRCA samples had mutations in the CRGs. Specifically, ATP7A had the highest mutation frequency (1%), followed by ATP7B, while others did not have any significant mutations (**Figure 2E**). Therefore, our findings on the landscape of CRGs in BRCA showed that they might play an essential role in the development and progression of BRCA.

### Identification of CuproptosisClusters in BRCA

**Figure 3:**
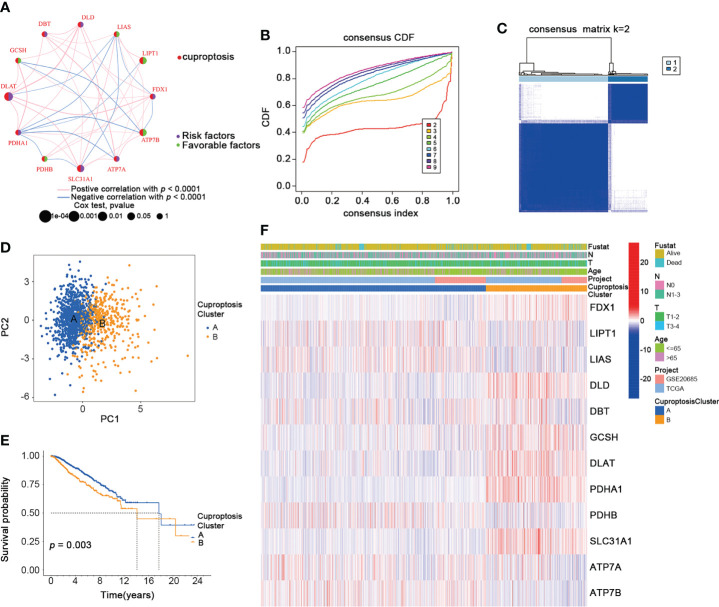
Identification of CuproptosisClusters in BRCA. **(A)** The interaction among CRGs in BRCA (green: favorable factors for overall survival; purple: risk factors for overall survival). **(B)** The relative change in area under consensus CDF curve for k=2 to 9. **(C)** The consensus clustering of BRCA patients for k = 2. **(D)** The PCA analysis of the two CuproptosisClusters. **(E)** The OS of the two CuproptosisClusters (Log-rank test). **(F)** The heatmap for the connections between clinicopathologic features and the two CuproptosisClusters (blue: low expression; red: high expression). CDF, cumulative distribution function; PCA, Principal component analysis; OS, overall survival. *p < 0.05, **p < 0.01, ***p < 0.001.

To further explore the expression pattern of CRGs implicated in BRCA tumorigenesis, we integrated patients from TCGA-BRCA (n = 1091) and GSE20685 (n = 327) as training cohort. First, the comprehensive landscape of CRG interactions and their prognostic value in the BRCA training cohort was demonstrated in a cuproptosis network (**Figure 3A**). We did the unsupervised clustering analysis using the “ConsensusClusterPlus” R package and picked k= 2 based on the empirical cumulative distribution function (CDF) plots, which suggested the highest intragroup correlations and the lowest intergroup correlations compared with others (**Figures 3B, C**, and **Figure S1**). Thus, two CRG-expression patterns were observed: CuproptosisCluster A and CuproptosisCluster B. Furthermore, the BRCA patients in the training cohort could be completely distinguished (**Figure 3D**). We also performed the Kaplan–Meier (K-M) survival analysis of the two clusters, suggesting a poor overall survival of patients in Cluster B (**Figure 3E**). Finally, we examined the clinical and pathological characteristics of the two BRCA clusters and the expression of the CRGs (**Figure 3F**).

### Characteristics of the TME in CuproptosisClusters of BRCA

**Figure 4:**
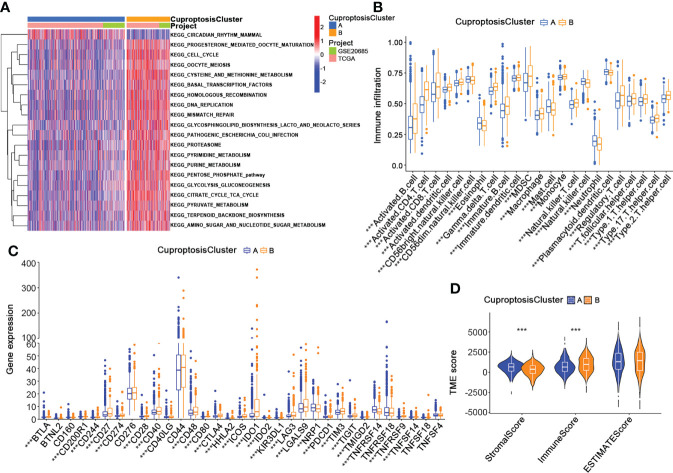
Characteristics of the TME in CuproptosisClusters of BRCA. **(A)** The GSVA of biological pathways between the two CuproptosisClusters (Spearman test, blue: inhibited pathways; red: activated pathways). **(B)** The abundance of 23 infiltrating immune cell types in the two CuproptosisClusters (Spearman test). **(C)** The expression levels of 32 immune checkpoints in the two CuproptosisClusters (Wilcoxon test). **(D)** The TME score of the two CuproptosisClusters (Spearman test). GSVA, gene set variation analysis; TME, tumor microenvironment. **p < 0.01, ***p < 0.001.

To thoroughly analyze the role of cuproptosis-related genes in the TME of BRCA, we conducted the GSVA enrichment analysis. As shown in **Figure 4A**, cluster A was significantly enriched in progesterone-mediated oocyte maturation, cell cycle, oocyte meiosis, cysteine and methionine metabolism, basal transcription factors, homologous recombination, DNA replication, mismatch repair, glycosphingolipid biosynthesis lacto and neolacto series, pathogenic escherichia coli infection, proteasome, pyruvate metabolism, pentose phosphate pathway, glycolysis gluconeogenesis, pyruvate metabolism, citrate cycle TCA cycle, terpenoid backbone biosynthesis, amino sugar and nucleotide sugar metabolism. Moreover, the results of the ssGSEA algorithm indicated that CuproptosisClusters A and B were rich in different innate immune cell infiltrations with significance (**Figure 4B**). We also examined the expression of 32 immune checkpoints in two clusters, which showed a higher expression of most immune checkpoints (BTLA, CDC20R1, CD244, CD27, CD274, CD28, CD40, CD40LG, CD48, CD80, CTLA4, HHLA2, ICOS, IDO1, IDO2, KIR3DL1, LAG3, LGALS9, PDCD1, TIGIT, TMIGD2, TNFRSF9, TNFSF14) in cluster B (**Figure 4C**). In addition, NRP1 and TNFRSF14 were highly expressed in cluster A. Using the “estimate” package, we evaluated the TME score in **Figure 4D**. Cluster A had a higher stromal score, while Cluster B had a higher immune score. According to the results above, we identified two clusters with distinct immunological and metabolic characteristics, suggesting that cuproptosis may affect the immune microenvironment and metabolic processes that lead to breast cancer progression.

### Identification of gene subtypes based on CuproptosisClusters of BRCA

**Figure 5:**
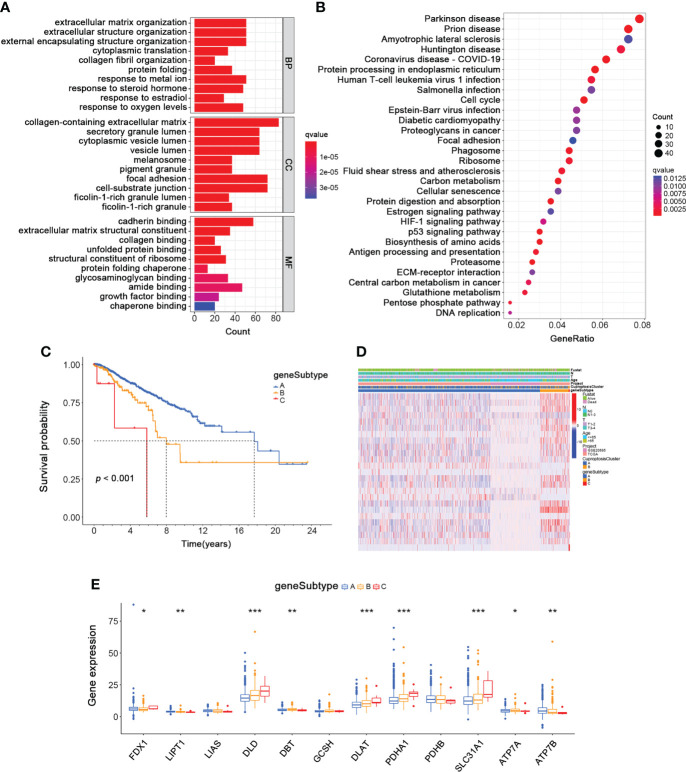
Identification of Gene Subtypes based on CuproptosisClusters of BRCA. **(A, B)** The GO and KEGG enrichment analyses of DEGs among the two CuproptosisClusters. **(C)** The overall survival of the three gene subtypes (Log-rank test). **(D)** The heatmap for the connections between clinicopathologic features and the three gene subtypes (blue: low expression; red: high expression). **(E)** The differences in the expression of 12 cuproptosis-related genes among the three gene subtypes (Wilcoxon test). DEGs, differentially expressed genes; GO, Gene Ontology; KEGG, Kyoto Encyclopedia of Genes and Genomes. *p < 0.05, **p < 0.01, ***p < 0.001.

Next, we identified 968 CuproptosisCluster-related DEGs by performing the “limma” package further to explore the different biological behaviors of each cluster (**Table S5**). Firstly, functional enrichment and GO (Gene Ontology) analysis and KEGG (Kyoto Encyclopedia of Genes and Genomes) pathway analysis were conducted among the CuproptosisCluster-related genes (**Figure 5A** **, B**, **Table S6** and **S7**). Then a univariable Cox regression analysis was performed to screen out 25 genes with significant prognostic values (*p < 0.001*) for the subsequent investigation (**Table S8**). Moreover, we applied the consensus to divide patients into three gene subtypes based on the 25 prognostic genes (**Figure S2**). As shown in **Figure 5C**, patients of gene subtype A showed the best OS, while patients of gene cluster C showed the worst OS (*p < 0.001*). The comparison of the clinicopathological characteristics and the expression of DEGs between the three gene subtypes was shown in the heatmap (**Figure 5D**). Finally, we observed the different expressions of the cuproptosis-related genes in the three gene subtypes (**Figure 5E**).

### Development of the cuproptosis-related gene signature and CRG_score in the BRCA training cohort

**Figure 6:**
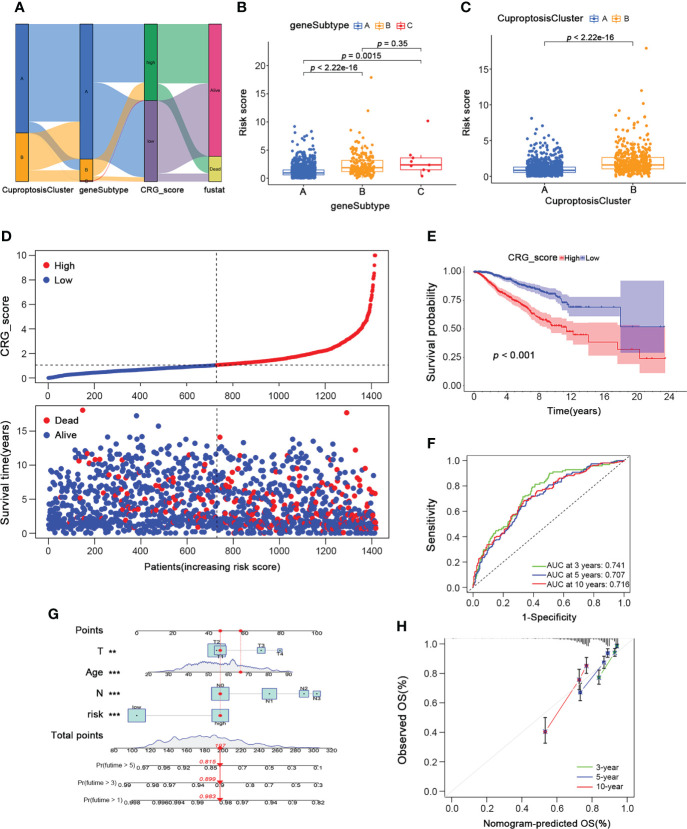
Development of the Cuproptosis-Related Gene Signature and CRG_score in the BRCA Training cohort. **(A)**The alluvial diagram showing the connection between CuproptosisClusters, gene subtypes, and CRG_score. **(B)** The level of CRG_score in the three gene subtypes (Wilcoxon test). **(C)** The level of CRG_score in the two CuproptosisClusters (Wilcoxon test). **(D)** The ranked dot and scatter plots of CRG_score distribution and patient survival status in BRCA training cohort. **(E)** The overall survival of the high and low CRG_score groups in BRCA training cohort (Log-rank test). **(F)** The ROC curves for the predictive efficiency of the CRG_score in BRCA training cohort (green: 3 year; blue: 5 year; red: 10 year). **(G)** The Nomogram to predict 3-, 5- and 10-year OS in the BRCA training cohort. **(H)** The Calibration plots of the nomogram to predict OS at 3-, 5- and 10-year (green: 3 year; blue: 5 year; red: 10 year). CRG_score, Cuproptosis-Related Gene Score; ROC, receiver operating characteristic. *p < 0.05, **p < 0.01, ***p < 0.001.

To further investigate the underlying mechanisms regulating breast cancer progression, we constructed a cuproptosis-related gene signature by performing the LASSO regression analysis based on the 25 prognostic subtype-related genes. And the Cuproptosis-Related Gene Score (CRG_score) was calculated as follows: CRG_score = (0.00523335734652904 * PGK1) + (0.0185186220360186 * SLC52A2) − (0.0261649532232335 * SEC14L2) + (0.0129284730002406 * RAD23B) − (0.0297061289632435 * SLC16A6) − (0.0142610013224485 * CCL5) + (0.0014557255170858 * MAL2). To better estimate the characteristics of patients with different levels of CRG_score, we divided the BRCA training cohort into high- and low-CRG_score groups depending on the median CRG_score. The alluvial diagram depicted the distribution of BRCA patients within the two cuproptosis clusters, three gene subtypes, and two CRG score groups (**Figure 6A**). As shown in **Figure 6B**, gene subtype C had significantly higher CRG_scores than the other two gene subtypes. Moreover, cuproptosis cluster B had higher CRG_scores than cluster A (**Figure 6C**). **Figure 6D** also showed the distribution plot of the survival of each BRCA patient from the training cohort, which indicated a higher death probability in the high-CRG_score group. The Kaplan-Meier curve consistently suggested a worse prognosis for the high-CRG_score group than the low-CRG_score group (*p < 0.001*; **Figure 6E**). Moreover, we performed the time-dependent receiver operating characteristic (ROC) analysis to calculate the AUC values of this cuproptosis-related gene signature (0.741 for 3-year, 0.707 for 5-year, and 0.716 for 10-year; **Figure 6F**). We also conducted a nomogram plot analysis, which suggested an excellent advantage for CRG_score in long-term survival prediction (**Figure 6G**). Finally, the calibration chart revealed an excellent performance of the CRG_score among the predicted and observed overall survival with a C index of 0.69 (**Figure 6H**).

### Validation of the cuproptosis-related gene signature in BRCA testing cohorts

**Figure 7:**
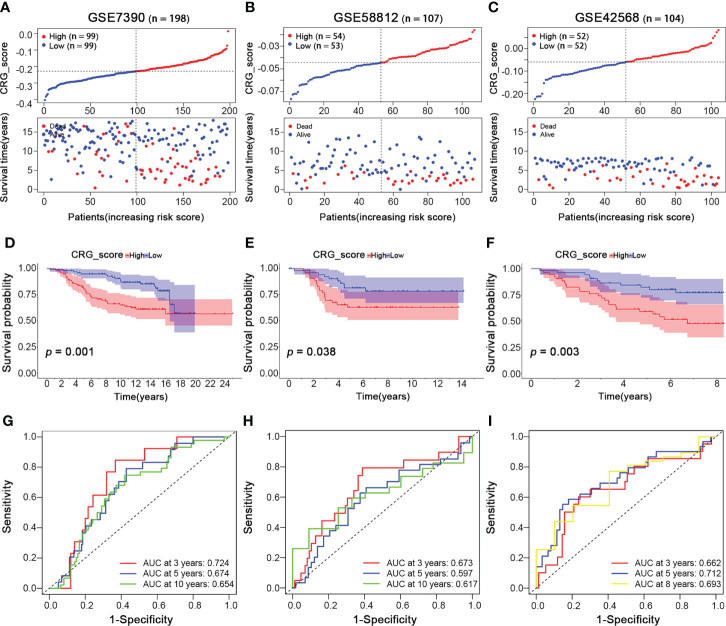
Validation of the Cuproptosis-Related Gene Signature in BRCA Testing Cohorts. BRCA Testing Cohorts: GSE7390 (n = 198), GSE58812 (n = 107), and GSE42568 (n = 104). **(A–C)** The ranked dot and scatter plots of CRG_score distribution and patient survival status in BRCA testing cohorts (GSE7390, GSE58812, and GSE42568 sets, respectively). **(D–F)** The overall survival of the high and low CRG_score groups in BRCA testing cohorts (GSE7390, GSE58812, and GSE42568 sets, respectively). **(G–I)** The ROC curves for the predictive efficiency of the CRG_score in BRCA training cohort (red: 3 year; blue: 5 year; yellow: 8 year; green: 10 year).

To validate the reliability and reproducibility of our cuproptosis-related gene signature, we calculated CRG_scores of three independent external validation BRCA groups, including GSE7390 with 198 BRCA patients, GSE58812 with 107 BRCA patients, and GSE42568 with 104 BRCA patients. Then the patients in each testing cohort were classified into high- and low-CRG_score groups based on the median CRG_score value. Patients with high CRG_scores in all three testing cohorts showed worse survival status (**Figures 7A** **–C**). Similarly, survival analysis revealed a significantly better overall survival of patients with low CRG_scores than those with high CRG_scores (*p = 0.001, 0.038, 0.003*, respectively; **Figure 7D** **–F**). Moreover, the high AUC values also suggested an excellent ability of the CRG_score to predict the long-term prognosis of BRCA patients in testing cohorts (**Figures 7G** **–I**). Thus, the results above showed a similar tendency to the training cohort, indicating the cuproptosis-related gene signature was stable and reliable.

Moreover, to further clarify the applicability of different molecular subtypes of breast cancer to our predictive gene signature, the PAM50 algorithm was conducted on the testing cohorts (GSE7390, GSE58812, and GSE42568; n = 409). The distribution of molecular subtypes according to the PAM50 signature was as follows: 13 normal-like (3%), 126 basal-like (31%), 111 luminal A (27%), 102 luminal B (25%), and 57 HER2+ (14%). We also calculated the CRG_scores of each PAM50 subtype and divided them into high- and low-CRG_score groups based on the median CRG_score value, respectively. The survival and ROC curve analyses were also performed for each PAM50 subtype (**Figure S3**). Patients of luminal A and HER2+ subtypes showed a better prognosis in the low CRG_score group (**Figures S3C, E**), while the survival of other subtypes was not statistically significant. In addition, the AUC values for all of the PAM50 subtypes were high, which indicated that our CRG_score could predict the long-term prognosis of BRCA patients with different molecular subtypes.

### Relationship of clinical characteristics and TME characteristics with CRG_score in the BRCA training cohort

**Figure 8:**
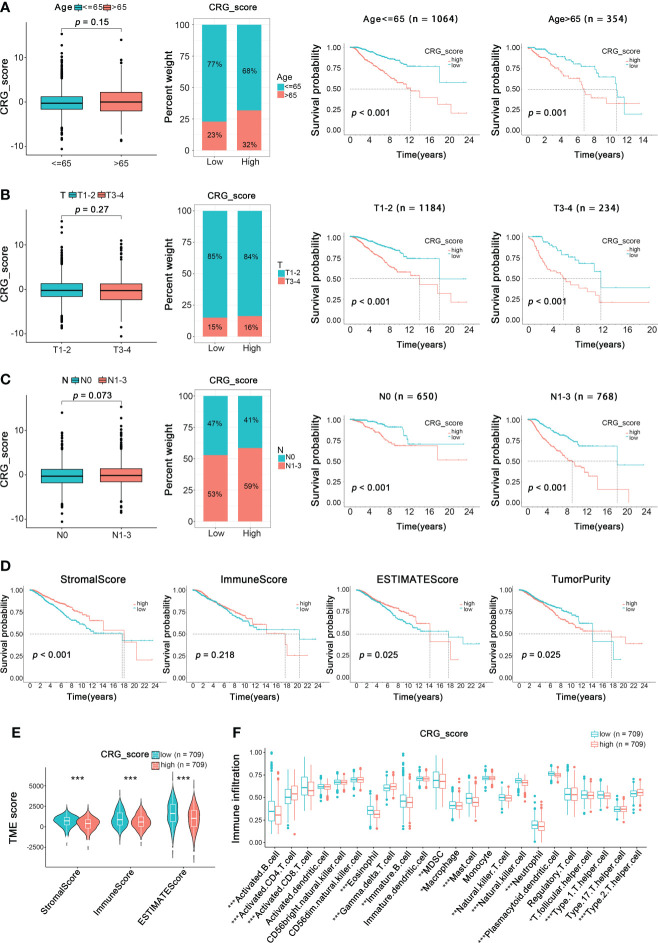
Relationship of Clinical Characteristics and TME Characteristics with CRG_score in the BRCA Training Cohort. The relationship between age **(A)**, T **(B)**, N **(C)** and CRG_score. The TNM system of cancer staging reflects the extent of tumor growth, where primary tumor (T), and nodal status for metastasis (N) (Wilcoxon test for boxplot; Log-rank test for survival analysis). **(D)** Survival analysis of ImmuneScore, StromalScore, ESTIMATEScore, and TumorPurity in BRCA patients (Log-rank test). **(E)** The TME score of the two CuproptosisClusters in the high and low CRG_score groups (Spearman test). **(F)** The abundance of 23 infiltrating immune cell types in the high and low CRG_score groups (Spearman test). *p < 0.05, **p < 0.01, ***p < 0.001.

Further, we investigated the clinical characteristics of the training cohort’s high- and low-CRG_score groups. For ages, patients aged ≥65 had a higher CRG_score, and the high CRG_score of patients of different ages was positively related to poor prognosis (**Figure 8A**). Then a relatively higher CRG_score was observed in patients with T1-2 and a lower CRG_score in patients with T3-4. In addition, the low CRG_scores of patients with different T stages suggested a better overall survival than the high CRG_score group (**Figure 8B**). Similarly, patients with N1-3 were correlated with a relatively high CRG_score, while the low CRG_score of patients with different N stages both lived longer than those in the high-CRG_score group (**Figure 8C**). Therefore, patients with a high CRG_score of types ≥65 age, stage T1-2, and stage N1-3 had worse long-term survival.

To analyze the relationship between TME and CRG_score, we calculated the TME score (ImmuneScore, StromalScore, ESTIMATEScore, and TumorPurity) using the “estimate” package (**Figure 8D**). We divided patients into low- and high-TME score groups depending on the median values. We found that low ImmuneScore and ESTIMATEScore were significantly correlated with poor overall survival (*p < 0.001*, *p = 0.025*, respectively), while high TumorPurity was significantly correlated with worse overall survival (*p=0.025*). Moreover, there was no significant correlation between the prognosis of BRCA patients and StromalScore. Besides, a low CRG_score was also significantly associated with ImmuneScore, StromalScore, and ESTIMATEScore (**Figure 8E**). Furthermore, the relationship between the cuproptosis-related gene signature and immune cell abundance was further estimated in box plots and scatter diagrams (**Figure 8F**). Here, the results above revealed a close relationship between the cuproptosis-related gene prognostic model and TME, which suggested the possibility of immunotherapy targeting cuproptosis-related genes to block BRCA progression.

### Prediction of immunotherapy response in high- and low-CRG_score groups in BRCA

**Figure 9:**
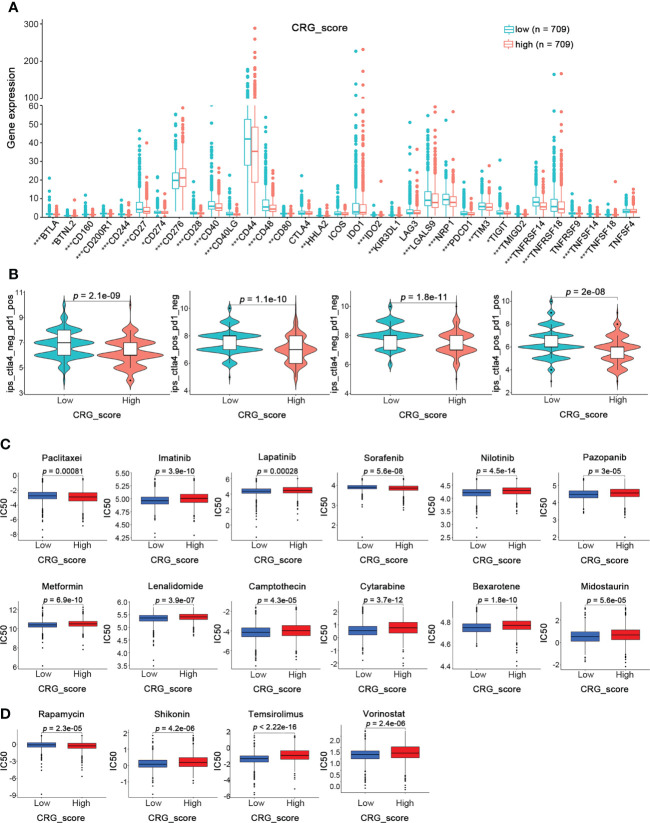
Prediction of Immunotherapy Response in High- and Low-CRG_score groups in BRCA. **(A)** The expression levels of 32 immune checkpoints in the high and low CRG_score groups (Wilcoxon test). **(B)** The immunotherapy response between the high and low CRG_score groups (Wilcoxon test). **(C)** The relationships between CRG_score and chemotherapeutic sensitivity (Wilcoxon test). *p < 0.05, **p < 0.01, ***p < 0.001.

Since cuproptosis played an essential role in the TME, we further investigated its influence on immunotherapy for BRCA. First, the expression of 32 critical immune checkpoints in high- and low-CRG_score groups was examined (**Figure 9A**). We observed that most immune checkpoints were significantly overexpressed in the low-CRG_score group. Next, we investigated the application of CRG_score in the therapy of BRCA. The BRCA immunotherapy profile of patients from the TCIA database revealed that the low-CRG_score group had higher ICI scores than the high-CRG_score group and was more responsive to the immunotherapy than the high-CRG_score group (**Figure 9B**). In addition, we assessed the responses of the high- and low-CRG_score groups to conventional and novel chemotherapeutic agents. The high-CRG_score group was more sensitive to lapatinib, nilotinib, pazopanib, metformin, lenalidomide, camptothecin, cytarabine, bexarotene, midostaurin, shikonin, temsirolimus, and vorinostat. In contrast, the low-CRG_score group was more responsive to paclitaxel, imatinib, sorafenib, and rapamycin (**Figure 9C**). Therefore, BRCA patients with high-CRG_score were characterized by abundant immune infiltration, high expression of immune checkpoints, and better response to immunotherapy and chemotherapy.

### Expressions pattern of cuproptosis-related gene signature in BRCA

**Figure 10:**
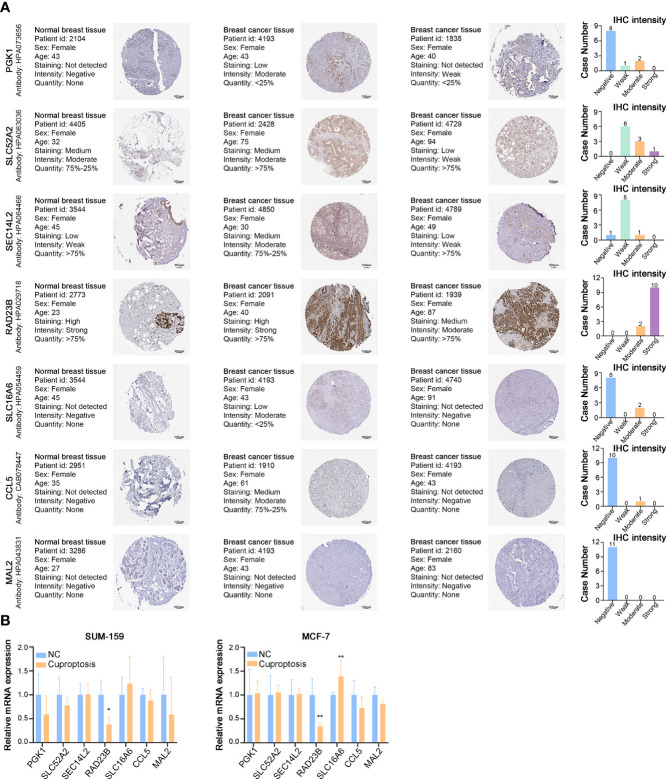
Expressions of Cuproptosis-Related Gene Signature in BRCA. **(A)** Representative IHC images of PGK1, SLC52A2, SEC14L2, RAD23B, SLC16A6, CCL5, and MAL2 across clinical specimens of normal and tumor samples in the Human Protein Atlas (HPA) database. Bar charts represent IHC staining intensities of PGK1 (11 patients), SLC52A2 (10 patients), SEC14L2 (10 patients), RAD23B (12 patients), SLC16A6 (10 patients), CCL5 (11 patients), and MAL2 (11 patients). **(B)** The relative mRNA expression of PGK1, SLC52A2, SEC14L2, RAD23B, SLC16A6, CCL5, and MAL2 in the cuproptosis cell model (RT-PCR). *p < 0.05, **p < 0.01.

To further explore the important role of the cuproptosis-related gene signature in breast cancer, we analyzed their protein expression patterns in the normal and tumor samples in the Human Protein Atlas (HPA) database (**Figure 10A**). RAD23B protein was strongly expressed in breast cancer tumor tissues, SLC52A2 was moderately expressed, and SEC14L2 was weakly expressed. Additionally, PGK1 was not expressed in most breast cancers, some were weakly expressed, and few were moderately expressed. As for SLC16A6, CCL5, and MAL2, their protein expression was almost negative in breast cancer tissues based on the results of the HPA database. According to the previous study (8), we also examined the transcriptional expression of our cuproptosis-related gene signature in the cuproptosis cell model. As shown in **Figure 10B**, RAD23B was significantly down-regulated in SUM159 and MCF7 cells after cuproptosis induction; SLC16A6 was significantly down-regulated in MCF7 cells, while other genes showed no significant change.

We also analyzed the mRNA expression of the cuproptosis-related gene signature in breast cancer tissues, suggesting a higher transcriptional expression of all 7 cuproptosis-related genes in BRCA tissues than in adjacent normal tissues (**Figure S4A**). Furthermore, overall survival analysis showed a worse prognosis for patients with high expression of PGK1, SLC52A2, RAD23B, and MAL2; while patients with up-regulated SEC14L2, SLC16A6, and CCL5 could live longer (**Figure S4B**).

### The role of RAD23B in the regulation of breast cancer progression, chemotherapy and immunotherapy *in vitro*

**Figure 11:**
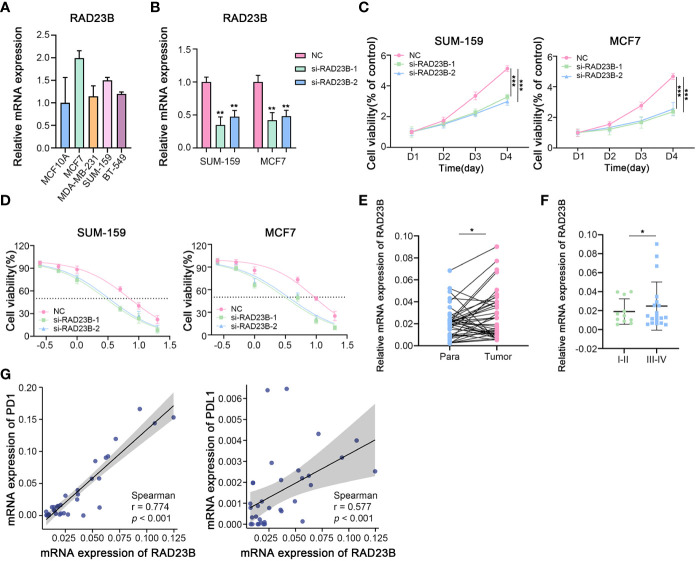
The Role of RAD23B in the Regulation of the Breast Cancer Progression, Chemotherapy and Immunotherapy *in Vitro*. **(A)** The mRNA expression levels of RAD23B in different breast cancer cell lines (RT-PCR). **(B)** The relative mRNA expression of RAD23B in SUM-159 and MCF7 breast cancer cells (RT-PCR; One-way ANOVA). **(C)** The cell viability assay after RAD23B reduction in SUM-159 and MCF7 breast cancer cells (MTT; Two-way ANOVA). **(D)** The IC50 of Paclitaxel after RAD23B reduction in SUM-159 and MCF7 breast cancer cells (MTT). **(E)** The relative mRNA expression of RAD23B in 34 pairs of breast cancer tissues and adjacent normal breast tissues (RT-PCR; Student’s t-test). **(F)** The relative mRNA expression of RAD23B in different pathologic stages of breast cancer tissues (RT-PCR; Student’s t-test). **(G)** Correlation between RAD23B mRNA expression and PD1/PDL1 mRNA expression (RT-PCR; Spearman test). *p < 0.05, **p < 0.01, ***p < 0.001.

The protein and mRNA expression levels of RAD23B were significantly higher in breast cancer tissues than in paraneoplastic tissues, and its expression was positively correlated with poor prognosis in BRCA patients. Moreover, the expression of RAD23B was decreased in the cuproptosis cell model, which indicated that it acted as a “risk” factor and antagonized cuproptosis in BRCA progression. Therefore, RAD23B was selected as a promising target for in-depth experimental validation. As shown in **Figure 11A**, the transcriptional expression of RAD23B was examined in breast epithelial cell lines (MCF10A), luminal breast cancer cells (MCF7), and triple-negative breast cancer cells (MDA-MB-231, SUM-159, and BT549). Due to the high expression of RAD23B in SUM-159 and MCF7 cells, two RAD23B siRNA (small interfering RNA) constructs were used to knockdown its expression in these two cell lines (**Figure 11B**). After the reduction of RAD23B, cell viability was significantly inhibited (**Figure 11C**). The MTT assay reduced the IC50 of Paclitaxel in the RAD23B down-regulated groups compared with that of the control group (**Figure 11D**). To further explore the expression pattern of RAD23B in breast cancer, we examined the mRNA expression level of RAD23B in 34 pairs of breast cancer tissues and adjacent normal breast tissues. It suggested that RAD23B was significantly overexpressed in breast cancer tissues than in adjacent normal breast tissues, and its expression was positively correlated with pathological grade (**Figure 11E** **, F**). Finally, we also tested the mRNA expression of PD1 and PDL1 in breast cancer tissues by performing real-time RT-PCR. Then the correlation between RAD23B and PD1/PDL1 was evaluated (**Figure 11F**; r = 0.774, *p < 0.001*; r = 0.577, *p < 0.001*, respectively).

## Discussion

Because of breast cancer’s high incidence and recurrence rates, its treatment has been an ongoing challenge for decades (4). Numerous studies have attempted to determine the significance of the immune microenvironment in breast cancer progression, and immunotherapy may be a viable treatment option for breast cancer patients (42–44). However, immunotherapy for breast cancer patients is still in its infancy, and more investigation is needed to benefit more people.

As an essential cofactor of key enzymes, copper must maintain a dynamic low concentration to maintain normal physiological activity. A few studies have noted the role of cooper in regulating cancer progression. It has been reported that the serum level of copper was significantly increased in the BRCA group compared to the control group, indicating its function in the early detection and monitoring of breast cancer (13, 24, 25). In triple-negative breast cancer (TNBC), inhibition of mitochondrial copper shifted tumor cells from respiration to glycolysis to reduce energy production, ultimately inhibiting tumor growth and improving prognosis (26–28). Recently reported, an excess load of intracellular copper could induce cell death termed cuproptosis (8). Independent of known cell death pathways, cuproptosis does not activate caspase-3 and cannot be blocked by apoptotic inhibitors (8). This study mainly discussed 12 cuproptosis-related genes, including FDX1, LIPT1, LIAS, DLD, DBT, GCSH, DLST, DLAT, and PDHA1, PDHB, SLC31A1, ATP7A, and ATP7B. These genes are primarily involved in processes such as glycolysis and the tricarboxylic acid (TCA) cycle (45, 46), steroids (47, 48), and vitamin D metabolism (49). SCL31A1 as a copper importer and ATP7A and ATP7B as copper exporters are essential for maintaining intracellular copper concentration (50, 51). As Peter et al. suggested, overexpression of SCL31A1 and deletion of ATP7B may increase susceptibility to cuproptosis (8). In addition, the knockout of nine genes (FDX1, LIAS, LIPT1, DLD, DLAT, PDHA1, PDHB, GCSH, and DBT) conferred resistance to cuproptosis (8). Although various studies have highlighted the significance of copper in breast cancer, there is a dearth of studies on the association between cuproptosis and breast cancer, mainly its function in the immune microenvironment and immunotherapy of breast cancer.

Our study first summarized the expression and mutation patterns of CRGs based on TCGA-BRCA and GSE20685. Although the frequency of global alterations was only 2.94%, all CRGs showed significant differences in expression and prognosis in BRCA samples compared to normal samples. Performing the unsupervised clustering algorithm, we classified breast cancer patients into two cuproptosis patterns (CuproptosisCluster A and CuproptosisCluster B). Compared to patients with Cluster A, those with Cluster B showed more advanced clinicopathological characteristics and a worse OS. A global examination of the TME for both clusters revealed that Cluster B presented enrichment in most immune cells and important immune checkpoints. In addition, Cluster A showed a higher stromal score with significance, while Cluster B had a higher immune score. Thus, the results above indicated that these two clusters were closely associated with TME in BRCA, implying a crucial role of CRGs in the immune regulation of breast cancer. Next, three cuproptosis-related gene subtypes were identified according to the DEGs of the two CuproptosisClusters. To further explore the role of cuproptosis in BRCA progression and TME, a cuproptosis-related gene signature (PGK1, SLC52A2, SEC14L2, RAD23B, SLC16A6, CCL5, and MAL2) and the CRG_score were constructed based on the training cohort and validated in the testing cohorts, as well as PAM50 subtyped testing cohorts. Furthermore, patients in high-CRG_score groups showed a worse prognosis under different clinicopathological features. And patients with a low-CRG_score exhibited enrichment in most immune cells and important immune checkpoints, and are more sensitive to immunotherapy. Also, a quantitative nomogram depending on CRG_score and tumor stage facilitated the prognostic stratification of breast cancer patients, further promoting the clinical application of CRG_score. In summary, the characteristics of TME differed significantly in the high- and low-CRG_score groups, suggesting CRGs could provide reasonable recommendations for personalized immunotherapy for breast cancer patients.

Regarding the cuproptosis-related gene signature, previous studies have identified the crucial roles of these seven genes in cell metabolism. PGK1, an ATP-generating enzyme, mediates mitochondrial metabolism and promotes tumorigenesis (52). SLC family genes such as SLC52A2 and SLC16A6 are important transporters in metabolic processes, and their dysregulation is associated with various diseases (53–56). SEC14L2 encodes lipid binding proteins and facilitates the uptake of Vitamin E (57). RAD23B is involved in nucleotide excision repair (NER) and is associated with cell apoptosis (58). CCL5, expressed and secreted by activated and normal T cells, could regulate the migration and chemotaxis of inflammatory cells (59–61). MAL2 has been reported to work as an essential component of the machinery for transcytosis in hepatoma HepG2 cells (62). However, there is still no research focus on the relationship between these seven genes and breast cancer cuproptosis. We then analyzed the protein expression of these seven genes in breast cancer tissues from the HPA database and their mRNA expression in the cuproptosis cell model. In particular, RAD23B was screened out for *in vitro* experimental validation. The results of RT-PCR, cell viability, and the IC50 assay illustrated that RAD23B expression was positively correlated with breast cancer progression, drug resistance, and poor prognosis in BRCA patients. More importantly, both PD1 and PDL1 were positively correlated with RAD23B, suggesting that patients with up-regulated RAD23B were more sensitive to immune checkpoint-blocking therapy targeting the PD-1/PD-L1 axis. Thus, our results confirmed the important role of cuproptosis in TME and immunotherapy for breast cancer, providing new ideas for immunotherapy with blocked immune checkpoints. Similarly, our cuproptosis-related scoring system was of great utility for clinical patient stratification, predicting the efficacy of adjuvant chemotherapy and patient prognosis. Also, our study laid an important research foundation for the role of cuproptosis in controlling the progression of breast cancer and made it easier to study its molecular mechanisms in more depth in the future.

Although we have performed a comprehensive analysis of cuproptosis in breast cancer and screened out potential targets to lay the foundation for future exploration of breast cancer progression, this study still has some limitations. Since our breast cancer samples are only obtained from retrospective studies from the TCGA and GEO databases, more cases from prospective research are required. In addition, experimental studies *in vivo* and *in vitro* are needed to validate our findings. Furthermore, additional research is necessary to identify the specific molecular mechanisms of cuproptosis regulating breast cancer progression.

## Data availability statement

The data sets analyzed during the current study are available in the TCGA (https://portal.gdc.cancer.gov/), accession numbers TCGA-BRCA, BRCA-FPKM; GEO repository (https://www.ncbi.nlm.nih.gov/geo/), accession numbers GSE20685, GSE7390, GSE58812, and GSE42568.

## Ethics statement

The studies involving human participants were reviewed and approved by The Ethics Committee on Human Research of the First Affiliated Hospital of Xi’an Jiaotong University. The patients/participants provided their written informed consent to participate in this study.

## Author contributions

SS designed the study and wrote the manuscript. MZ and PX analyzed data and contributed to writing the manuscript. SW and YW revised the manuscript. All authors contributed to the article and approved the submitted version.

## Funding

This research was funded by the National Natural Science Foundation of China (No. 82173365), the Key projects of Natural Science Foundation of Shaanxi Province(2021JZ-36); Natural Science Basic Research Project of Shaanxi Province (2020JM-362); the Institutional foundation of The First Affiliated Hospital of Xi’an Jiaotong University (No.2020QN-34).

## Conflict of interest

The authors declare that the research was conducted in the absence of any commercial or financial relationships that could be construed as a potential conflict of interest.

## Publisher’s note

All claims expressed in this article are solely those of the authors and do not necessarily represent those of their affiliated organizations, or those of the publisher, the editors and the reviewers. Any product that may be evaluated in this article, or claim that may be made by its manufacturer, is not guaranteed or endorsed by the publisher.

## Supplementary material

Supplementary Figure 1 PCA analysis for the construction of CuproptosisClusters.

Supplementary Figure 2 PCA analysis for the construction of Gene Subtypes.

Supplementary Figure 3 Validation of the cuproptosis-related gene signature in different BRCA subtypes of testing cohorts.

Supplementary Figure 4 The survival analysis and expression of Cuproptosis-related gene signature in BRCA.
