# Supplementary figures and images for: Comprehensive analysis of cuproptosis-related genes and tumor microenvironment infiltration characterization in breast cancer
Source: Front Immunol. 2022 Oct 20;13:978909. doi: 10.3389/fimmu.2022.978909 (PMC9630583; doi:10.3389/fimmu.2022.978909)

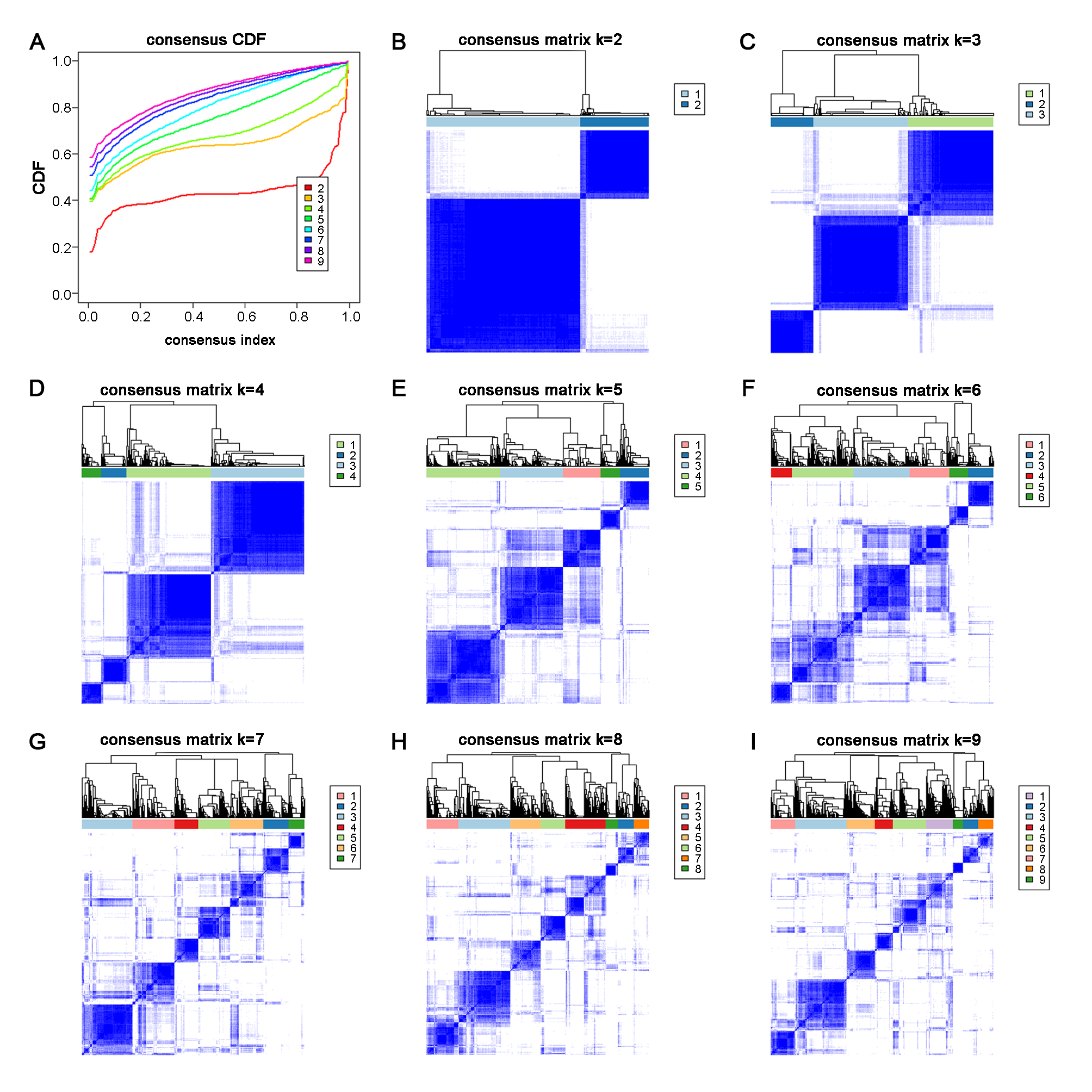

Supplement: Supplementary Figure 1 — PCA analysis for the construction of CuproptosisClusters. [file Image_1.tif]

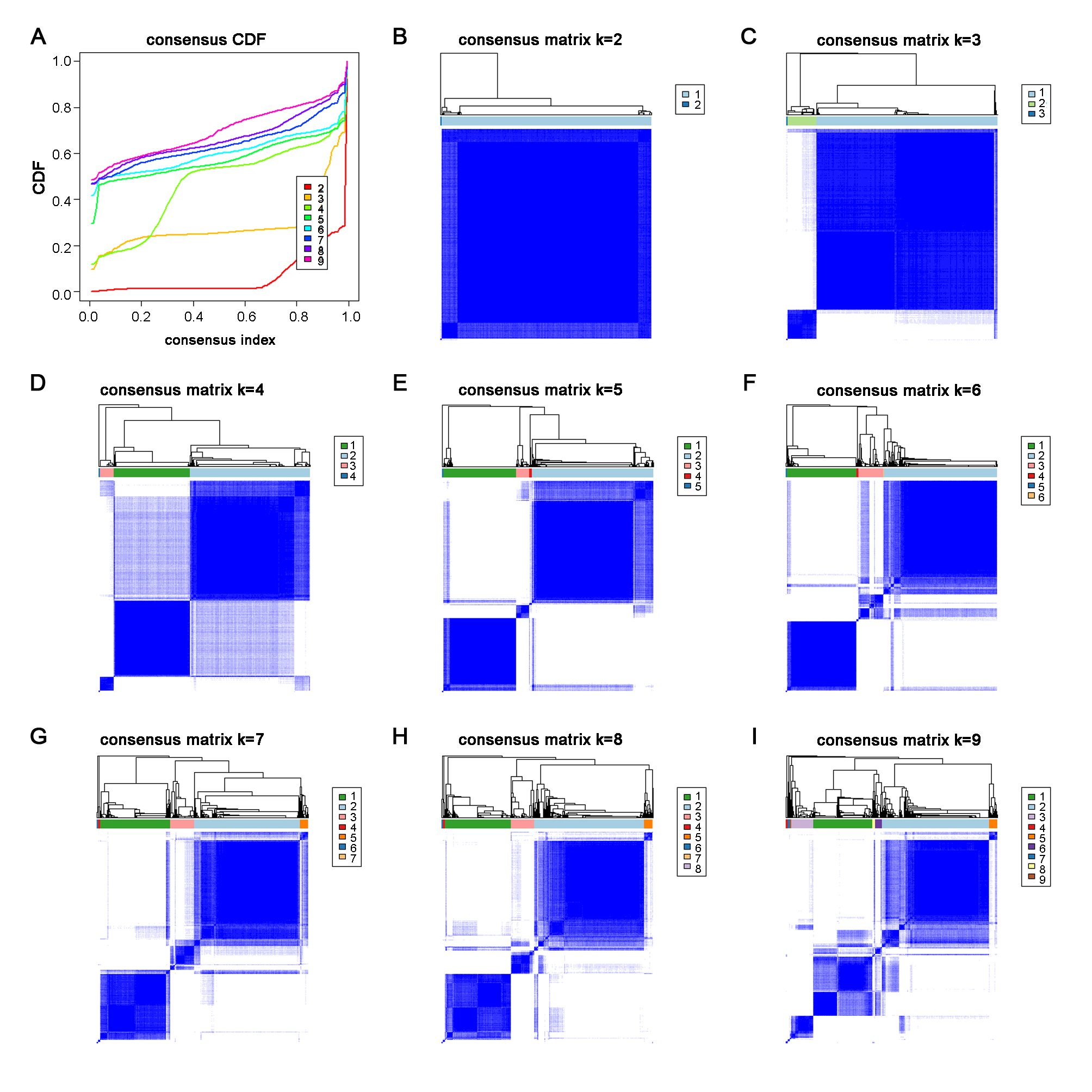

Supplement: Supplementary Figure 2 — PCA analysis for the construction of Gene Subtypes. [file Image_2.tif]

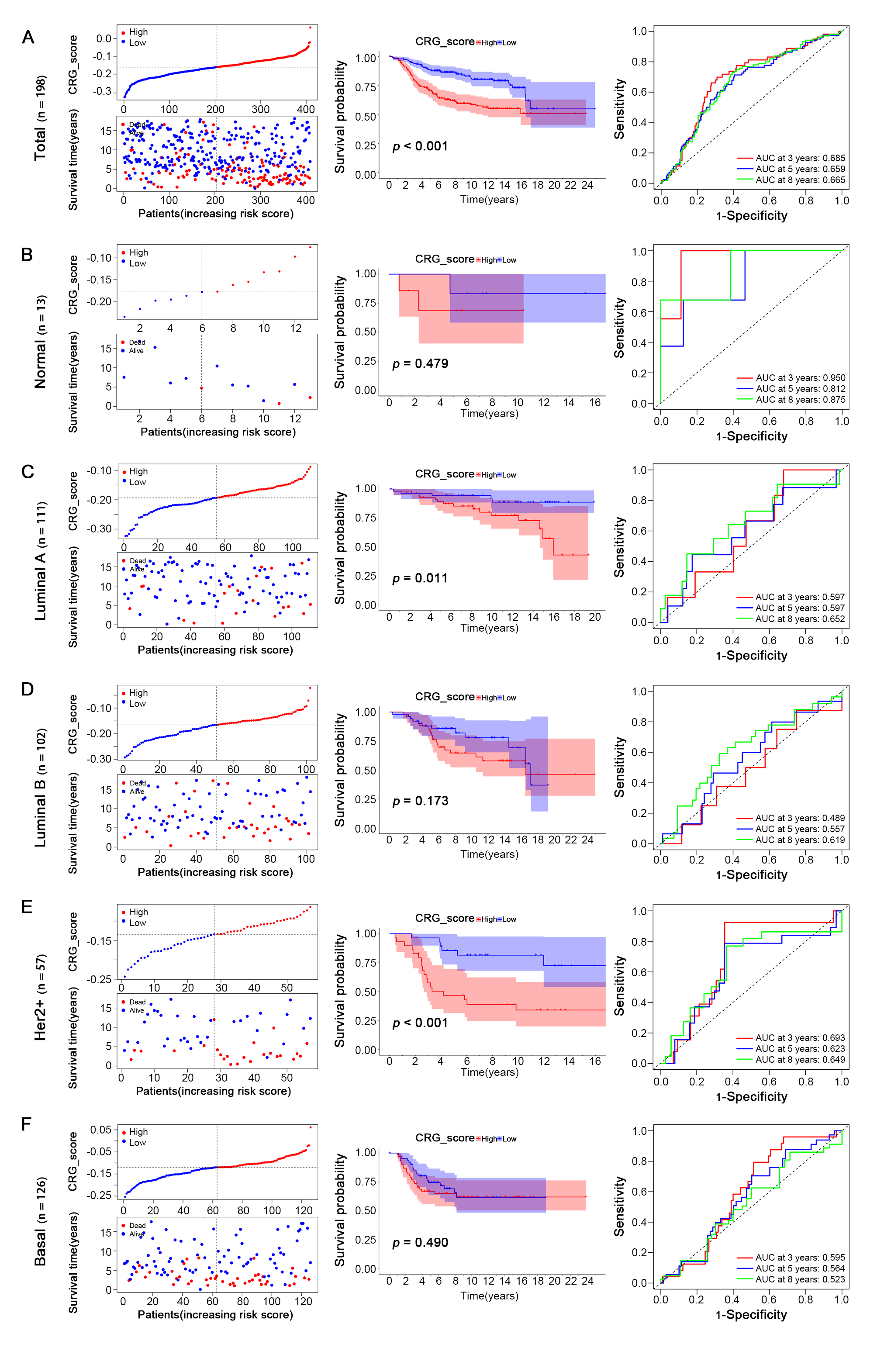

Supplement: Supplementary Figure 3 — Validation of the cuproptosis-related gene signature in different BRCA subtypes of testing cohorts. [file Image_3.tif]

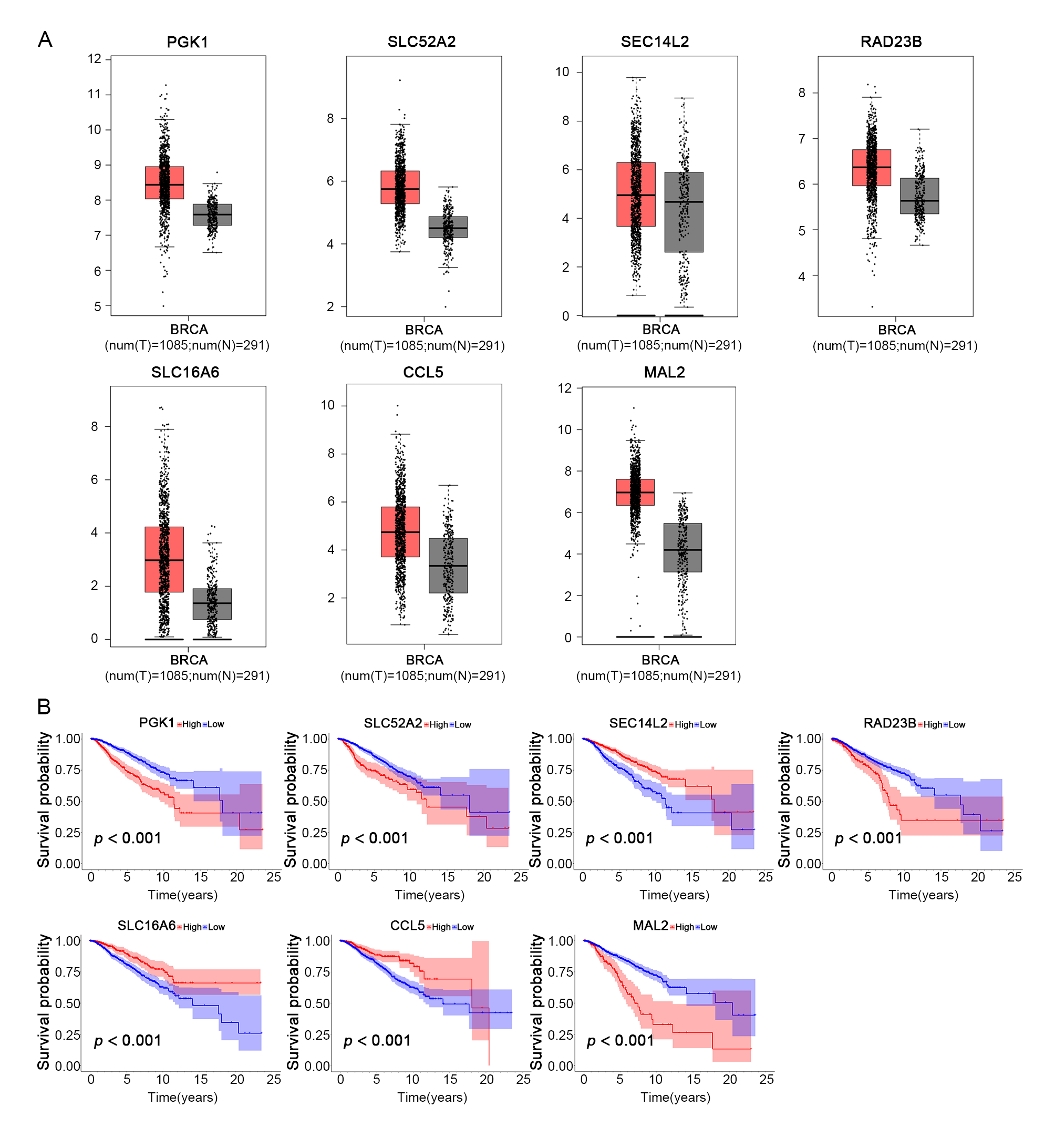

Supplement: Supplementary Figure 4 — The survival analysis and expression of Cuproptosis-related gene signature in BRCA. [file Image_4.tif]
